# Evaluation of precipitation from CMORPH, GPCP-2, TRMM 3B43, GPCC, and ITPCAS with ground-based measurements in the Qinling-Daba Mountains, China

**DOI:** 10.1371/journal.pone.0185147

**Published:** 2017-10-02

**Authors:** Gefei Wang, Peiyun Zhang, Liwen Liang, Shiqiang Zhang

**Affiliations:** 1 Shaanxi Key Laboratory of Earth Surface System and Environmental Carrying Capacity, Northwest University, Xi’an, Shaanxi Province, China; 2 College of Urban and Environmental Science, Northwest University, Xi’an, Shaanxi Province, China; Columbia University, UNITED STATES

## Abstract

The correspondence between five precipitation products, including CMORPH, GPCP-2, TRMM 3B43, GPCC, and ITPCAS, and ground-based measurements of precipitation were evaluated on annual, seasonal, and monthly scales during 2000–2014 in the Qinling-Daba Mountains over China, which is a significant area with vital value of climate and hydrology. Performances of the precipitation products in the relatively arid/humid years were also analyzed. In general, ITPCAS data displayed the highest accuracy, GPCP-2 and CMORPH data showed relatively poor performance, and GPCC and TRMM 3B43 data were average at different temporal scales among the five precipitation products. The Pearson correlation coefficient of each station had minor fluctuations for the five precipitation products. A larger deviation was found at Wudu station, most likely due to the undulating terrain. The performances of the precipitation products from highest to least accuracy are as follows: ITPCAS > TRMM 3B43 > GPCC > GPCP-2 > CMORPH. Except for CMORPH (-20.76%), the percentage precipitation differences (PPDs) of the other four precipitation products fluctuated in the range of 10% during the relatively arid (2001) and humid (2011) years. In addition, all precipitation products and ground gauge observed precipitation did not show an obvious gradient with altitude, which is different from that in other mountainous areas and is perhaps due to complex terrain, lack of observation in high altitudes, and precipitation undercatch. In consideration of the significance of Qinling-Daba Mountains as the geographic and ecological dividing lines, the present study may provide a new perspective for hydrological, climatic, and ecological researches and practices in local and other mountainous areas.

## Introduction

Precipitation is widely recognized as a fundamental component of the global water cycle and plays a critical role in the survival of human beings as our primary source of fresh water. It also has both direct and indirect economic impacts on human activities [1]. Information on the rates, amounts, distribution, among other factors of precipitation is indispensable for a wide range of applications, including agronomy, hydrology, meteorology, and climatology [2]. A rain gauge is a mechanical and simple ground-based measurement tool for rainfall, which has high accuracy on a point or local scale. However, since rain gauges are limited in local areas on land with uneven distribution, it is hard to acquire accurate global and regional distribution of precipitation using these devices.

Satellites offer an incomparable advantage to observe Earth’s system processes and parameters [3]. With the improvement of remote sensing and its correction technology, precipitation distribution on global and regional scales can be obtained from satellite precipitation products with high spatial resolution and continuous time series. Many precipitation products used to access climate information, including CMORPH (Climate Prediction Center morphing technique), GPCP-2 (Global Precipitation Climatology Project), TRMM 3B43 (Tropical Rainfall Measuring Mission), GPCC (Global Precipitation Climatology Center), Dataset of ITPCAS (Institute of Tibetan Plateau Research, Chinese Academy of Sciences), PERSIANN (Precipitation Estimation from Remote Sensing Information using an Artificial Neural Network), and GSMaP (Global Satellite Mapping of Precipitation), have been developed. Nevertheless, satellite precipitation products need to be evaluated against in situ observations and then calibrated to various hydrological models before application to hydrological operations [4]. It should also be noted that the performance of different precipitation products seems to have various effects on different regions.

Dinku et al. [5] reported that TRMM-3B43 and 3B42, CMAP (the Climate Prediction Center Merged Analysis of Precipitation), CMORPH, and TAMSAT (Tropical Applications of Meteorology using SATellite and other data) performed reasonably well in East Africa. Romilly and Gebremichael [6] pointed out that TMPA 3B42RT (Real Time) and CMORPH outperformed PERSIANN overall over an Ethiopian river basin, which tended to underestimate rainfall by 43%, a much larger percentage than that of CMORPH and TRMM 3B42RT. Yamamoto et al. [7] evaluated TMPA, CMORPH, PERSIANN, and GSMaP with gauge data for the Khumb Region in the Nepal Himalayas and discovered an increase in precipitation during the summer monsoon and during the morning for all evaluated precipitation products except GSMaP. Chen et al. [8] found that JRA-55 (the 55-yr Japanese Reanalysis Project), ERA-Interim (ECWMF Re-Analysis Interim), NCEP CFSR (Climate Forecast System Reanalysis), and NASA MERRA (Modern-Era Retrospective Analysis of Research and Applications) were consistent in reproducing the interannual variability of rainfall diurnal cycles in East Asia, and JRA-55 gave a good capture of the early-evening rainfall over the Tibetan Plateau. Shen et al. [9] reported that PERSIANN, NRL (Naval Research Laboratory blended product), TRMM 3B42, TRMM 3B42RT, CMORPH, and the arithmetic mean of the microwave estimates used in CMORPH were able to capture the overall spatial distribution and temporal variations in China and directly pointed out that the performance of satellite products varied for different precipitation regimes and regions. All studies mentioned above indicate that the evaluation of different precipitation products in different regions is significant and necessary for obtaining accurate information on precipitation in certain regions.

In addition, Janowiak et al. [10] compared NCEP-1 reanalysis precipitation with GPCP data over the period 1988–1995 and suggested that good agreements occurred for large-scale features, but substantial differences existed on regional scales. Huffman et al. [11] and Kummerow et al. [12] pointed out the necessities for adequate validations on regional scales instead of using global approaches. Zhao and Fu [13] found that ERA-40 and NCEP-2 were able to reflect the temporal and spatial distribution of precipitation but showed regional variation after preliminarily comparison with observation data. Ma et al. [14] evaluated precipitation from ERA-40, NCEP-1, NCEP-2, CMAP-1, CMAP-2, and GPCP-2 with ground-based measurements in China and determined that CMAP-1 and GPCP-2 had better correspondence with adjusted observational precipitation in general. However, the validity of these conclusions on a regional scale is still unclear, especially in mountain regions with complex terrain.

The Qinling-Daba Mountains, referring to both Qinling Mountain and Daba Mountain, are the division lines of geography and climate between northern and southern China, which are also roughly consistent with 0°C isothermal curves (north of this line usually below 0°C, south of this line above 0°C) in January, 800 mm isohyet curves, and 2000 h sunshine hour contour lines [15, 16]. The Qinling-Daba Mountains serve as an important water source for the Middle Route of South-to-North Water Diversion Project in China. Although these mountains are significant regions for resourcing water and have pivotal meteorological and hydrological significance to the whole area, validation and assessment of precipitation products in the area are lacking. While numerous precipitation products are available on a global scale, we chose 5 for our study. As the first stage of evaluation, the performances of CMORPH, GPCP-2, TRMM 3B43, GPCC, and ITPCAS on annual, seasonal, and monthly scales were evaluated in the Qinling-Daba Mountains, which may provide a new perspective and guidance for water resource management, climate change research, and hydrological modeling in this and other regions. This paper is organized as follows: Section 2 describes the study area; Section 3 introduces the datasets, evaluation indexes, and methodology used; Section 4 describes the results of evaluating precipitation products on annual, seasonal, monthly scales and at individual rain gauge stations; Section 5 discusses the performances over the relatively arid and humid years and precipitation gradients; and the final section presents the conclusions.

## Study area

**Fig 1:**
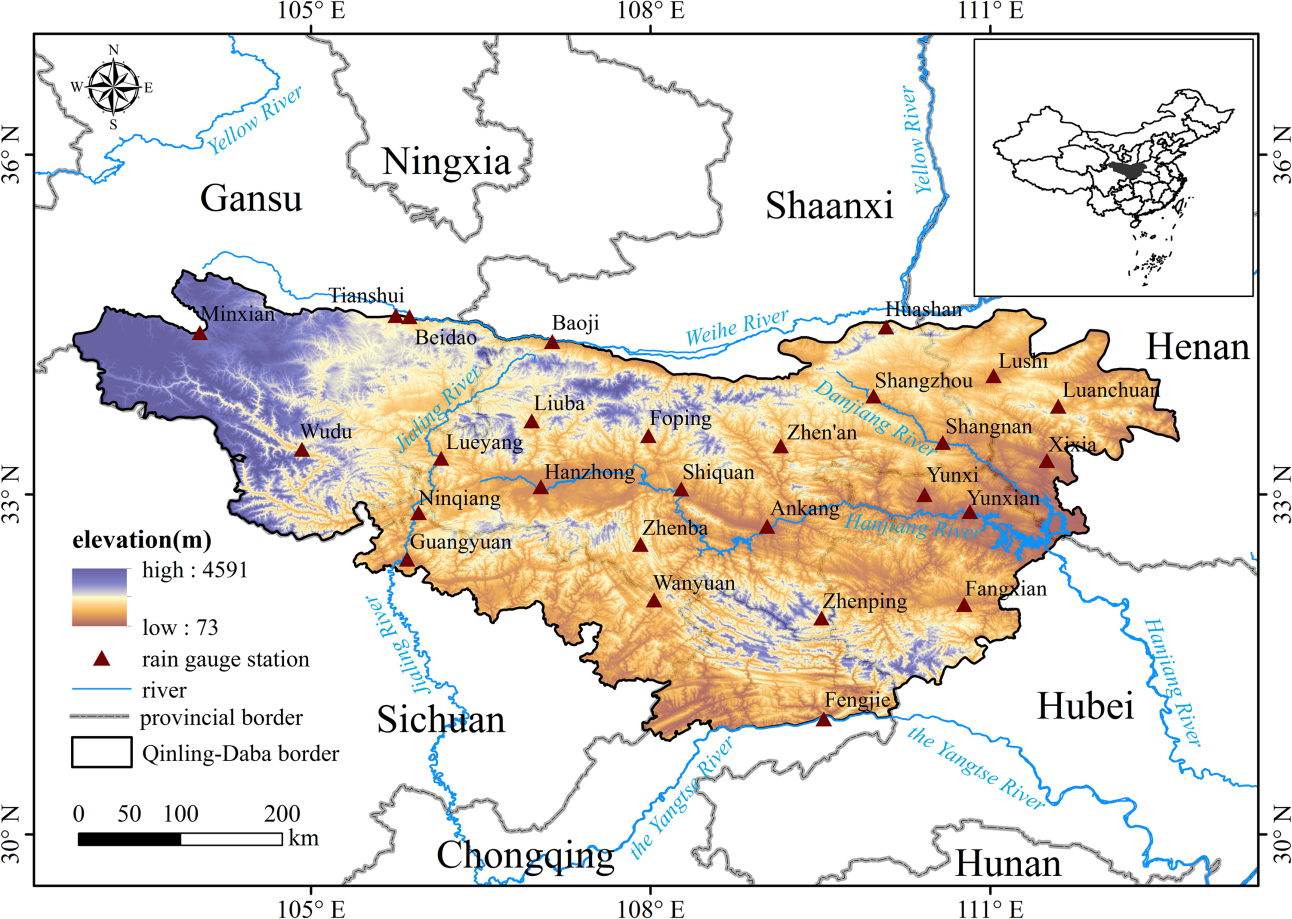
Location of the Qinling-Daba Mountains and the distribution of rain gauge stations.

**Fig 2:**
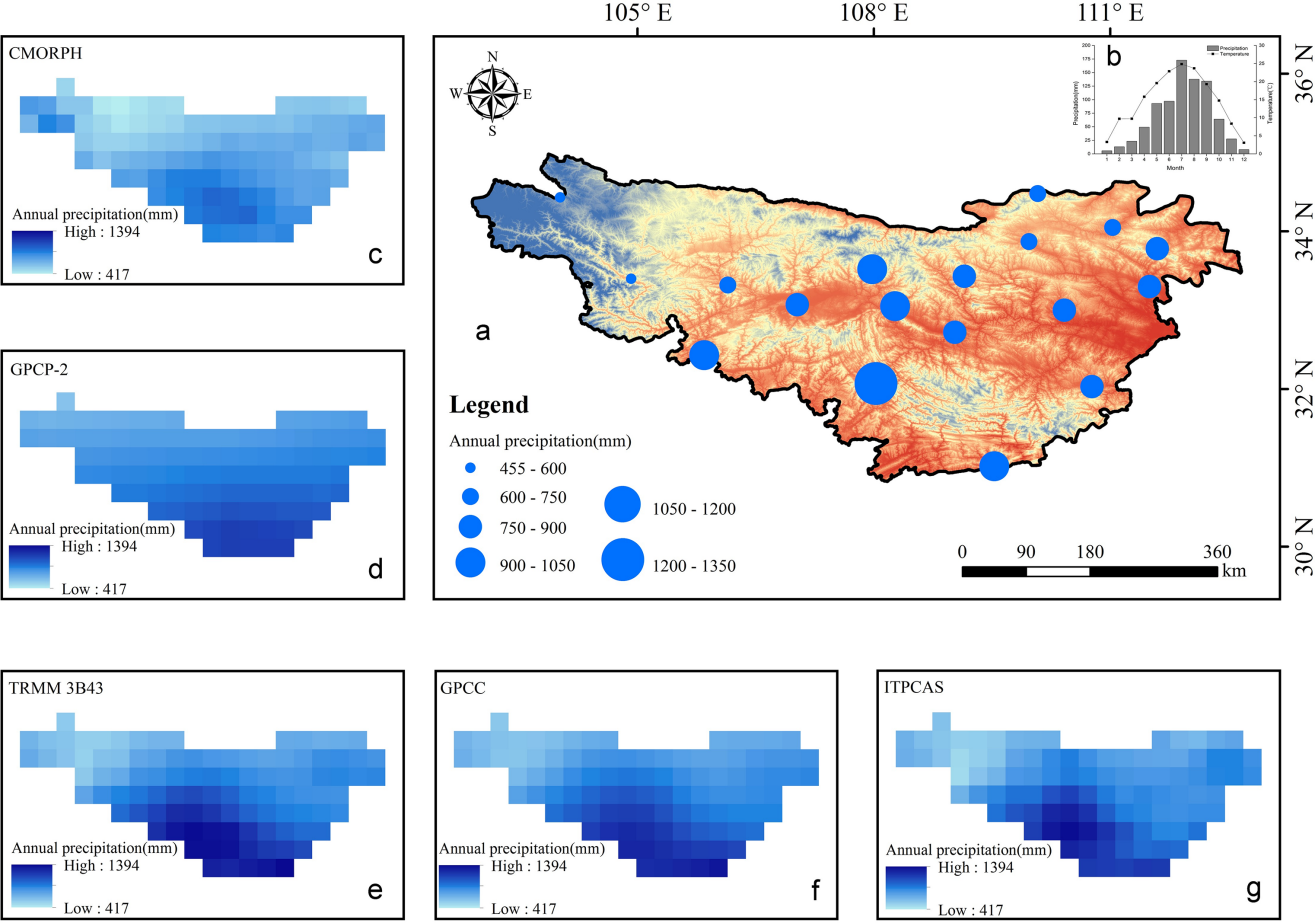
The basic climate pattern in the Qinling-Daba Mountains. (a) The spatial distribution of annual rainfall from ground-based measurements. (b) The seasonal distribution of rainfall and temperature, the spatial distribution of annual rainfall from five precipitation products including (c) CMORPH, (d) GPCP-2, (e) TRMM 3B43, (f) GPCC, and (g) ITPCAS.

The Qinling-Daba Mountains are located in central China (30°50′-34°59′N, 102°54′-112°40′E) and span a total area of about 222,300 km^2^ (Fig 1) [17, 18]. According to the Digital Elevation Model (DEM) in Fig 1, the difference between the maximum and minimum elevation is about 4000 m in these mountains. The basic spatial distribution of annual precipitation (Fig 2A), which is displayed by colored and sized meteorological sites, suggests the annual precipitation in the northwestern region is about 500 mm and is far less than that in the southwestern areas (about 1300 mm). At the division lines of geography and climate between northern and southern China, precipitation in the Qinling-Daba Mountains is distributed with significant spatial and temporal heterogeneity due to the transitional climate and drastic altitude changes. From Fig 2B, it is obvious that the Qinling-Daba Mountains possess four distinct season patterns, where precipitation mainly occurs during summer and autumn. The spatial distribution of mean annual rainfall from precipitation products is shown in Fig 2C–2G, where TRMM 3B43, GPCC, and ITPCAS are more consistent with ground-based measurement than CMORPH and GPCP-2 compared to Fig 2A.

## Data and methodology

### Data

#### Precipitation products

Precipitation products, including CMORPH, GPCP-2, TRMM 3B43, GPCC, and ITPCAS, were evaluated in this study. The first four precipitation datasets with different resolutions represent various retrieval algorithms, and ITPCAS was created as a reanalysis product for China and has been testified in multiple domains and regions [19–21]. The five precipitation products were used to discuss the influence of various resolutions, rain gauge stations, among other parameters. A summary of the various precipitation products is given in Table 1, and a brief description of each product is given below.

**Table 1:** List of precipitation products evaluated in this study.

Product	Temporal resolution	Spatial resolution(°)	Methodology
CMORPH	half-hourly	0.07277×0.07277	Aggregated to monthly, seasonal and annual precipitation
GPCP-2	Monthly	2.5×2.5	Aggregated to seasonal and annual precipitation
TRMM 3B43	Monthly	0.25×0.25	Aggregated to seasonal and annual precipitation
GPCC	Monthly	1.0×1.0	Corrected and aggregated to seasonal and annual precipitation
ITPCAS	Monthly	0.1×0.1	Aggregated to seasonal and annual precipitation

CMORPH, a satellite-only product, is produced through a morphing technique that uses precipitation estimates from passive microwave observations and propagates these features using motion vectors from geostationary satellite IR (infrared) imagery at half-hour intervals [22]. The shape and intensity of the precipitation features are modified during the time between microwave sensors scans by performing a time-weighted linear interpolation [23]. For the global precipitation products with the finest resolution in this paper, CMORPH with a 0.07277° latitude/longitude resolution (8 km × 8 km at the equator) was obtained for analysis every 30 min between 60°S and 60°N (http://www.cpc.ncep.noaa.gov/products/janowiak/cmorph_description.html).

GPCP-2 has the coarsest resolution compared to the other four precipitation products analyzed in this study. It incorporates precipitation estimates derived from low-orbit microwave data, geosynchronous infrared data, and surface rain gauge observations. GPCP Version1 is a combination of precipitation estimates from MW (microwave) and IR (infrared) sensors in polar orbit satellites and geostationary satellites, as well as surface observations. GPCP-2 calibrates, or adjusts, the more frequent geosynchronous infrared observations by utilizing the higher accuracy of the low-orbit microwave observations [24]. GPCP-2 is better developed compared to GPCP Version1 due to the additions of TOVS (TIROS (Television and Infrared Observation Satellite) Operational Vertical Sounder) and AIRS (Atmospheric Infrared Sounder) estimates, OLR (outgoing longwave radiation) measurements, and Global Historical Climate Network and Climate Assessment and Monitoring System [25]. GPCP-2 data in a 2.5° × 2.5° grid was obtained from NOAA (https://www.esrl.noaa.gov/psd/data/gridded/data.gpcp.html) [26].

TRMM 3B43 is a standard monthly precipitation product [27] that incorporates the combination of precipitation datasets, including TMI (TRMM Microwave Imager), PR (Precipitation Radar), and VIRS (Visible and Infrared Scanner), with SSM/I (Special Sensor Microwave Imager) and rain gauge data [28, 29]. TRMM 3B43 is derived by averaging the TRMM 3B42 V6 precipitation products and is widely used in climatological applications. It also provides estimates of the total monthly rainfall recorded from 1998 to present day with a spatial resolution of 0.25° and can be obtained from NASA (https://mirador.gsfc.nasa.gov/cgi-bin/mirador/presentNavigation.pl?project=TRMM&tree=project).

The GPCC’s (Global Precipitation Climatology Centre) Monitoring Product Version5 in a 1.0° × 1.0° grid with a timespan from 1982 to present was obtained for analysis from the following website (ftp://ftp.dwd.de/pub/data/gpcc/html/gpcc_monitoring_v5_doi_download.html) [30]. It is based on SYNOP and monthly CLIMAT reports received via GTS (Global Telecommunication System) of WMO (World Meteorological Organization) from 7000–8000 stations (after automatic and manual quality control) and is generated within 2 months after the end of the observation month. Sophisticated quality control and harmonization of the station metadata is crucial for GPCC in merging various datasets to detect errors in the station meta information and to ensure consistency of time series. Monthly rainfall data are obtained at GPCC from monthly totals calculated from SYNOP reports received at DWD and NOAA and a combination of the monthly CLIMAT reports received at various RTHs (Regional Telecommunication Hubs), which are interpolated and merged by GPCC to improve the spatial coverage and data quality [31].

The dataset of ITPCAS was developed by the Institute of Tibetan Plateau Research, Chinese Academy of Sciences (http://dam.itpcas.ac.cn/rs/?q=data) [32, 33]. It presents a reanalyzed dataset with a continuous time series and high spatial and temporal resolutions (0.1° × 0.1°, 3 h) that contains seven elements, including precipitation rate, pressure, et al [34]. The ITPCAS data used in this paper contain precipitation rates on a monthly scale.

The preliminary precipitation product of ITPCAS was produced following the subsequently described steps (1–4). 1) Gridded data merged from TRMM 3B42 and GLDAS-1 were interpolated into the 756 meteorological stations. 2) The differences between the observations and the interpolated TRMM 3B42/GLDAS-1 values (output of step1) were calculated at each station point. 3) The difference values (output of step2) were interpolated back into grid points, forming a so-called “correction field”. 4) This field was added to the TRMM 3B42/GLDAS-1 field as a manner of bias-correction [21]. Although the biases in TRMM 3B42/GLDAS-1 were corrected using the observed data at each time step, the cumulative rainfall over a month or higher time scale were often found to be overestimated because of the unrealistic rainfall produced by spatial interpolation. To overcome this problem, the observed monthly rainfall of 756 stations and similar steps as stated above were combined to control the biases in preliminary precipitation products on time scales over a month or longer as a way to obtain complete precipitation products.

#### Ground-based measurement data

Daily precipitation records from 27 rain gauge stations in the Qinling-Daba Mountains over 2000–2014 were collected from the National Meteorological Information Center of the China Meteorological Administration (CMA, http://data.cma.cn/). Manual quality control for rainfall data from ground-based station was performed. All stations are distributed in low and middle altitudes from 200 to 2300 m, and scarce stations are distributed in areas with elevations over 3000 m. Among the stations, 9 had complete records for several years but without a continuous time series. Therefore, 27 stations were used to evaluate annual and monthly precipitation, while 18 stations were evaluated for seasonal precipitation, the individual station, and precipitation gradient. Information about the rain gauge stations is summarized in Table 2.

**Table 2:** The information about rain gauge stations.

Number	Name	Latitude	Longitude	Elevation/m	Record period
56093	Minxian	34.43	104.02	2315	2000–2014
56096	Wudu	33.4	104.92	1079	2000–2014
57006	Tianshui	34.58	105.75	1142	2000–2003,2007–2008
57014	Beidao	34.57	105.87	1085	2004–2014
57016	Baoji	34.35	107.13	612	2000–2004,2007–2008
57046	Huashan	34.48	110.08	2065	2000–2014
57067	Lushi	34.05	111.03	569	2000–2014
57077	Luanchuan	33.78	111.6	750	2000–2014
57106	Lueyang	33.32	106.15	794	2000–2014
57124	Liuba	33.65	106.95	1547	2009–2014
57127	Hanzhong	33.07	107.03	510	2000–2014
57134	Foping	33.52	107.98	827	2000–2014
57143	Shangzhou	33.87	109.97	742	2000–2014
57144	Zhen’an	33.43	109.15	694	2000–2014
57154	Shangnan	33.46	110.58	1137	2009–2014
57156	Xixia	33.3	111.5	250	2000–2014
57206	Guangyuan	32.43	105.85	514	2000–2014
57211	Ningqiang	32.84	105.95	1400	2009–2014
57232	Shiquan	33.05	108.27	485	2000–2014
57237	Wanyuan	32.07	108.03	674	2000–2014
57238	Zhenba	32.56	107.91	1231	2009–2014
57245	Ankang	32.72	109.03	291	2000–2014
57251	Yunxi	33	110.42	249	2000–2014
57253	Yunxian	32.85	110.82	202	2007–2008
57259	Fangxian	32.03	110.77	427	2000–2014
57343	Zhenping	31.91	109.51	1615	2009–2014
57348	Fengjie	31.02	109.53	300	2000–2014

Despite the limited range and intrinsic error of rain gauge stations, they remain the most direct and precise measurement tool for precipitation so far [35]. Thus, ground-based measurements were considered as “true precipitation” datasets for reference in this research [36].

#### DEM

DEM is a representation of terrain elevation as a function of geographic location [37], which is widely used in digital terrain analysis, soil and water conservation, and related geographic researches. SRTM (Shuttle Radar Topography Mission) is an international project conducted by NASA and the National Geospatial-Intelligence Agency (NGA) to generate near-global land elevation products. The most commonly used SRTM DEM V4.1 was released by the Consortium for Spatial Information of the Consultative Group of International Agricultural Research (CGIAR-CSI) with a spatial resolution of 90 m [38] (http://srtm.csi.cgiar.org), which was adopted in this work to show the basic elevation variation in the Qinling-Daba Mountains.

### Methodology

#### Data preprocessing

For proper evaluation and comparison of precipitation data from different sources, a uniform spatial and temporal resolution was first acquired to place all precipitation products under the same conditions [14, 39].

In consideration of the base temporal resolution of GPCP-2, TRMM 3B43, GPCC Monitoring Product and ITPCAS, evaluation was conducted on a monthly scale and also on seasonal and annual scales aggregated from monthly data.

In order to compare the five products under the same conditions, all precipitation products were resampled to the same horizontal 0.5° × 0.5° grid scale to guarantee the coherent spatial resolution. Bilinear interpolation was adopted by combining the surrounding four grids [40], considering that it is a popular method for meteorological estimation [41, 42]. There are only 18 continuous stations over the Qinling-Daba Mountains with great variations in altitude, so they cannot be used to grid the ground-based measurements. Since there was only one rain gauge station in the 0.5° × 0.5° grid box, the rainfall data derived from precipitation products nearest to a station’s location were compared with the rainfall data from the same ground station. The rain gauge station in that grid box represented the observed rainfall amount corresponding to simulated rainfall from precipitation products in that grid for subsequent comparison [36, 43].

Data of the five precipitation products, including CMORPH, TRMM 3B43, GPCP-2, GPCC, and ITPCAS, were aggregated to season and annual accumulation. GPCC was adjusted by a correction factor provided by metadata before the aggregate was conducted. Additionally, a buffer of 0.1° was utilized to ensure that the rain gauge stations in the study area depicted a more accurate distribution of precipitation (considering the boundary effect).

The two methods employed to investigate the performances of five precipitation products over the relative arid and humid years included PD (absolute precipitation difference) and PPD (percentage of precipitation difference):

$$PD=P_{p}-P_{g}$$ (1)

$$PPD=\frac{P_{p}-P_{g}}{P_{g}}\times 100\%$$ (2)

where *P*_*p*_ is the rainfall value of a precipitation product; and *P*_*g*_ is the rainfall value from ground-based measurements.

#### Evaluation indexes

A series of indexes were used to quantitatively assess the performances of precipitation products with ground-based measurements from different perspectives. These included the Pearson correlation coefficient (r), root mean square error (RMSE), mean absolute error (MAE), and relative bias (BIAS).

$$r=\frac{\sum_{i=1}^{n}\left(G_{i}-\bar{G}\right)\left(P_{i}-\bar{P}\right)}{\sqrt{\sum_{i=1}^{n}{\left(G_{i}-\bar{G}\right)}^{2}{\left(P_{i}-\bar{P}\right)}^{2}}}$$ (3)

$$BIAS=\frac{\sum_{i=1}^{n}\left(P_{i}-G_{i}\right)}{\sum_{i=1}^{n}G_{i}}\times 100\%$$ (4)

$$RMSE=\sqrt{\frac{1}{n}\sum_{i=1}^{n}{\left(G_{i}-P_{i}\right)}^{2}}$$ (5)

$$MAE=\frac{1}{n}\sum_{i=1}^{n}\left|G_{i}-P_{i}\right|$$ (6)

where *n* is the total number of samples of gauge and satellite-based precipitation; *G* is the observation precipitation of ground-based measurements; *P* is the rainfall value from precipitation products in corresponding time intervals; $\bar{G}$ and $\bar{P}$ are the average values over *n* of *G* and *P*, respectively; and *r* is used to assess the linear agreement between rain gauge precipitation and precipitation products. A Range of -1 to 1 with positive values indicates positive correlation, while negative values represent negative correlation. *RMSE* indicates the overall level of error, which is sensitive to large or small errors. Positive and negative errors are considered errors in *MAE* to assess the accuracy of data. *BIAS* reflects the extent of deviation of precipitation products in rainfall values, in which the closer to 0 the *BIAS* is, the more accurate the precipitation product is.

Given the importance of the correlation coefficient to evaluate the precipitation products emphasized in a large number of studies [44–46], the correlation coefficient (r) was adopted as a primary and principal evaluation index to quantitatively assess the degree of correlation between observed precipitation and corresponding precipitation products to define the simulation accuracy in this study. Rigorous significance tests of correlation coefficients were done through T-test and discussed in the following sections. After that, BIAS was analyzed to determine if overestimation or underestimation existed in the simulation of precipitation products. Finally, RMSE and MAE were used to further determine the specific error amount in precipitation products under different circumstances.

## Evaluation of precipitation products

### Annual precipitation

**Fig 3:**
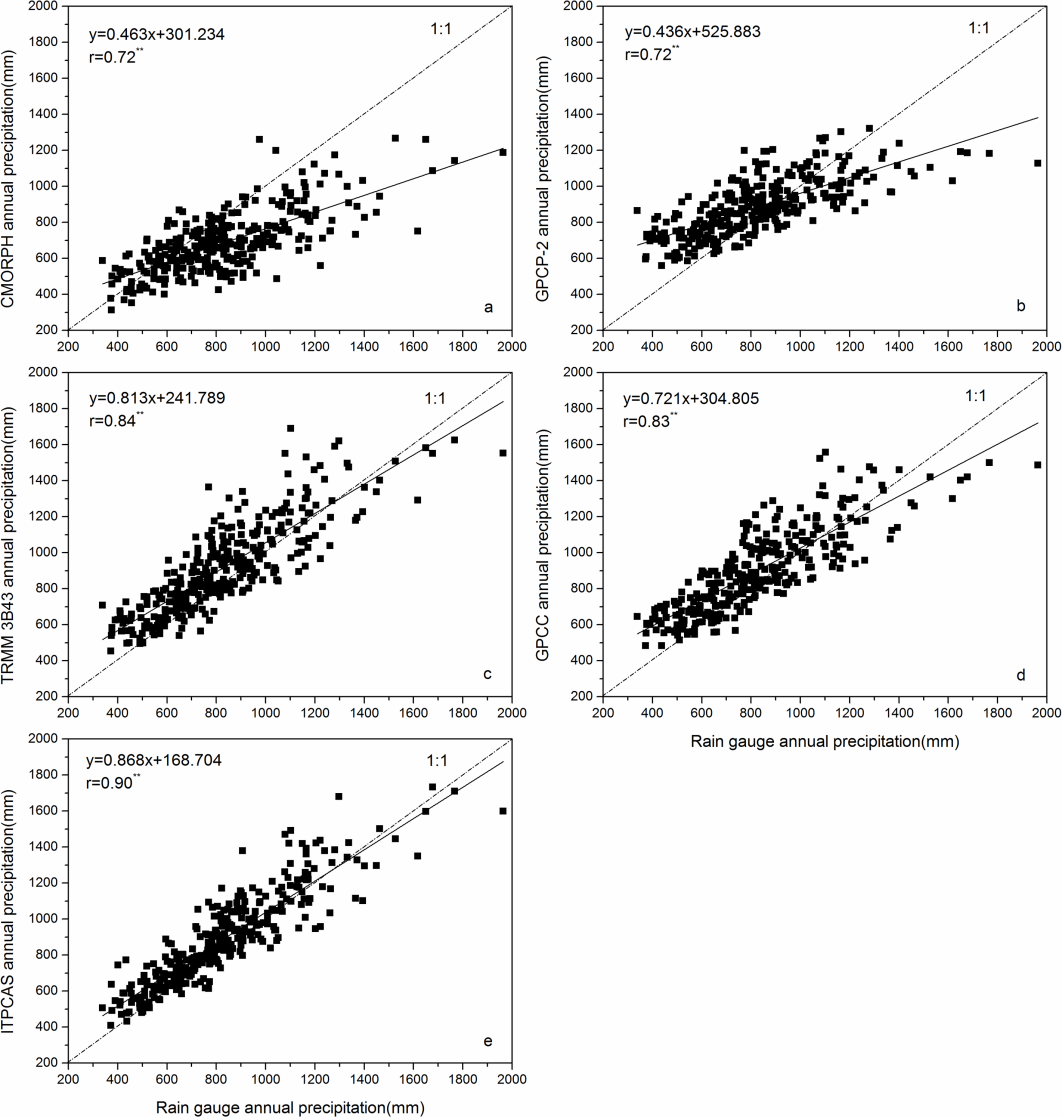
**Scatter plots of annual precipitation between precipitation products including (a) CMORPH, (b) GPCP-2, (c) TRMM 3B43, (d) GPCC, (e) ITPCAS, and corresponding rain gauge data during 2000–2014** (no asterisk beside the r value denoted this product failed to pass the significance test at the 95% confidence level; one asterisk beside the r denoted this product has passed the significance test at the 95% confidence level; two asterisks denoted this product has passed the significance test at the 99% confidence level on an annual scale).

To compare the annual precipitation from CMORPH, GPCP-2, TRMM 3B43, GPCC, and ITPCAS with ground-based measurements, scatter plots of annual rainfall from precipitation products versus rain gauge data were created and shown in Fig 3.

It is clear that TRMM 3B43, GPCC, and ITPCAS had high values of r above 0.8, which indicate that these three products, especially ITPCAS with an r value of 0.90, had high agreement with rain gauge observations on an annual scale. CMORPH and GPCP-2 possessed almost equal r values of 0.72. TRMM 3B43 and GPCC exhibited the similar skills for rainfall estimation based on r values of 0.84 and 0.83, respectively.

The other indexes are listed in Table 3. CMORPH had an underestimation of about -17.33% (the largest BIAS of the five precipitation products), while GPCP-2, TRMM 3B43, GPCC, and ITPCAS overestimated annual precipitation by about 7.02%, 10.52%, 8.87%, and 7.19%, respectively.

**Table 3:** BIAS, RMSE, and MAE of annual precipitation between five precipitation products and corresponding rain gauge data during 2000–2014.

Index	CMORPH	GPCP-2	TRMM 3B43	GPCC	ITPCAS
BIAS/%	-17.33	7.02	10.52	8.87	7.19
RMSE/mm	231.48	191.76	168.65	165.02	130.40
MAE/mm	178.24	151.27	132.81	133.29	97.75

Meanwhile, ITPCAS exhibited the least RMSE with a value of 130.40, which means it had the smallest discrepancy with the observation rainfall. In comparison to GPCP-2 and CMORPH, GPCC and TRMM 3B43 showed smaller and similar RMSE values of 165.02 and 168.65, respectively. The lowest and highest MAE values occurred in ITPCAS and CMORPH.

The above comparisons reveal that the correspondence with rain gauge data of ITPCAS was better than the other evaluated products, although it had a slightly larger BIAS than GPCP-2 on an annual scale. After that, TRMM 3B43 and GPCC had an almost equal degree of agreements with observed data for similar evaluation indexes. There is no doubt that GPCP-2 and CMORPH agreed the least, where CMORPH had the smallest r value and the greatest RMSE, MAE, and BIAS values.

### Seasonal precipitation

**Fig 4:**
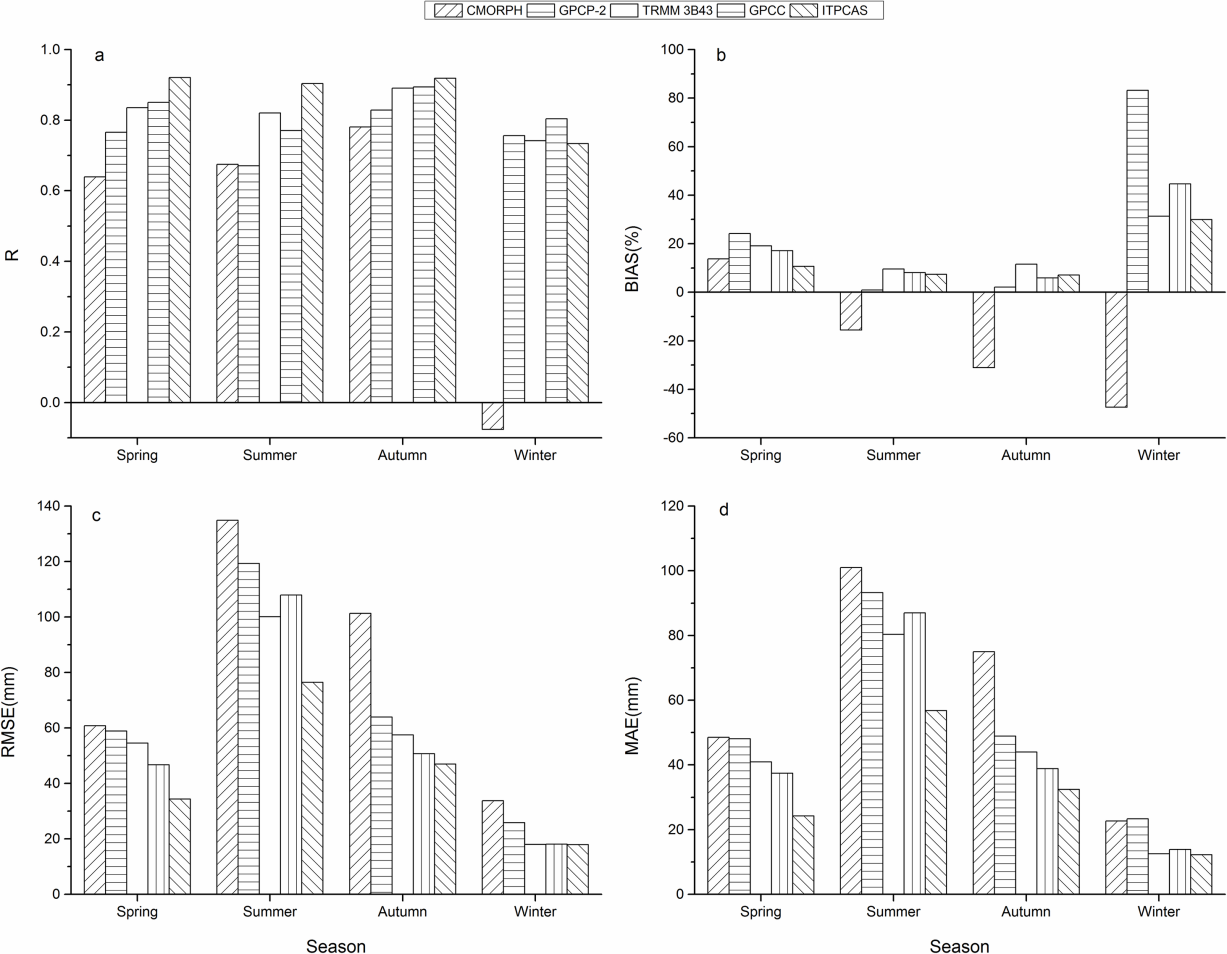
**Evaluation indexes between seasonal precipitation from precipitation products and corresponding rain gauge data in various seasons during 2000–2014 including (a)R, (b)BIAS, (c)RMSE, (d)MAE**.

To capture the full season, March-May are denoted as the spring months, June-August summer months, September-November autumn months, and December-following February winter months. Values of r, RMSE, MAE, and BIAS of precipitation products from the different seasons are shown in Fig 4.

Except CMORPH with an r value of -0.08 in winter, r values of the other four precipitation products were above 0.6 (Fig 4A), which suggests good agreement with ground-based measurements on a seasonal scale. In addition, CMORPH failed to pass the significance test at the 95% confidence level in winter, while the other products passed the significance test at the 99% confidence level (including CMORPH in spring, summer, and autumn). Almost all products had the best performance in autumn out the four seasons, which is most likely related to the uniform and abundant rainfall throughout the autumn months. CMORPH, TRMM 3B43, and ITPCAS performed the poorest in winter. Furthermore, it is worth mentioning that ITPCAS displayed the top representation for rainfall in spring, summer, and autumn among all products, while a smaller r value of 0.73 occurred in winter for ITPCAS compared to GPCP-2, TRMM 3B43, and GPCC. CMORPH exhibited the worst performance in spring, autumn, and winter with a performance in winter too poor to simulate the true rainfall. Except for CMORPH in spring, summer, and winter and GPCP-2 in summer, the other products performed fairly well.

The BIAS for TRMM 3B43, GPCC, and ITPCAS were within 50% (Fig 4B), and ranged from 0.87% to 83.25% for GPCP-2 and from -47.41% to 13.73% for CMORPH in the four seasons. GPCP-2 showed the largest BIAS, with the largest overestimation of 83.25% in winter. Meanwhile, CMORPH had a huge underestimation in all seasons except spring, with a maximum bias close to -50% in winter. The other products overestimated seasonal rainfall for all seasons in varying degrees. All precipitation products displayed a larger relative deviation in winter, perhaps due to the uneven and scarce precipitation in the Qinling-Daba Mountains during those months. ITPCAS had a slightly higher BIAS than GPCC in autumn but the lowest BIAS value among all precipitation products during the other three seasons.

Precipitation products displayed a similar trend for RMSE and MAE (Fig 4C and 4D) with higher values in summer and lower values in winter, which were consistent with the order of seasonal precipitation amounts. For summer and winter, TRMM 3B43 had RMSE values of 100.11 and 18.00, which were slightly lower than that of GPCC, 107.92 and 18.12, respectively. In spring and autumn, GPCC had lower RMSE values, 46.73 and 50.66, compared to those of TRMM 3B43, 54.49 and 57.48, respectively. Similar results occurred for MAE values between TRMM 3B43 and GPCC, which can be inferred that they have a similar amount of error on a seasonal scale.

Overall, ITPCAS had the highest r value, lowest RMSE and MAE values, and a relatively low BIAS, which indicates better agreement with the rain gauge observations on a seasonal scale compared to the other products. Contrarily, GPCP-2 and CMORPH displayed comparatively bad performances in the seasonal precipitation evaluation among the five precipitation products, while TRMM 3B43 and GPCC showed average and similar performances.

### Monthly precipitation

Taking into account the possible impact of the seasonal cycle on the correlation coefficients, monthly precipitation was stratified per month. After that, twelve correlation coefficients between the five precipitation products and ground-based measurements were calculated (Table 4). All precipitation products passed the significance test in all months, except CMORPH in December, which was consistent with the performance of CMORPH on a seasonal scale.

**Table 4:** R values between precipitation products and corresponding rain gauge data within twelve months (no asterisk denoted this product failed to pass the significance test at the 95% confidence level; one asterisk denoted this product passed the significance test at the 95% confidence level; two asterisks denoted this product passed the significance test at the 99% confidence level).

Month	CMORPH	GPCP-2	TRMM 3B43	GPCC	ITPCAS
January	-0.14*	0.69**	0.71**	0.80**	0.76**
February	0.38**	0.66**	0.69**	0.75**	0.69**
March	0.38**	0.73**	0.72**	0.77**	0.82**
April	0.62**	0.71**	0.80**	0.81**	0.92**
May	0.59**	0.77**	0.83**	0.84**	0.90**
June	0.65**	0.67**	0.80**	0.78**	0.89**
July	0.66**	0.70**	0.84**	0.79**	0.92**
August	0.49**	0.64**	0.77**	0.74**	0.84**
September	0.75**	0.80**	0.87**	0.87**	0.91**
October	0.64**	0.75**	0.86**	0.89**	0.89**
November	0.70**	0.84**	0.87**	0.90**	0.87**
December	-0.11	0.79**	0.79**	0.87**	0.77**
Average	0.47	0.73	0.80	0.82	0.85

The average correlation coefficients of the five precipitation products on a monthly scale were lower than that on an annual scale, except for GPCP-2, which had rigorous correlation coefficients that were considered as the performance of precipitation products on a monthly scale after removing the possible effects of seasonal cycle to a certain degree.

The highest r value for ITPCAS and lowest r value for CMORPH were shown in Table 4, while the r values for the other three products ranged between these two.

GPCP-2, TRMM 3B43, GPCC, and ITPCAS had overestimations with BIAS values of 7.02%, 10.52%, 8.87%, and 7.19%, respectively (Table 5). In contrast, CMORPH had the largest underestimation of about -17.33%.

**Table 5:** BIAS, RMSE, and MAE of monthly precipitation between five precipitation products and corresponding rain gauge data during 2000–2014.

Index	CMORPH	GPCP-2	TRMM 3B43	GPCC	ITPCAS
BIAS/%	-17.33	7.02	10.52	8.87	7.19
RMSE/mm	47.74	39.50	31.62	32.64	25.22
MAE/mm	28.78	24.52	19.58	19.79	14.62

ITPCAS had a lower RMSE value of 25.22 compared to the other products (Table 5). The RMSE value of 31.62 for TRMM 3B43 was slightly smaller than GPCC’s value of 32.64. Meanwhile, GPCP-2 performed worse than ITPCAS, TRMM 3B43, and GPCC with an RMSE value of 39.50. CMORPH owned the largest RMSE value of 47.74. The MAE had the similar trend with RMSE (Table 5).

In summary, ITPCAS displayed perfect r, RMSE, and MAE values and a relatively low BIAS value of 7.19%, which is just slightly higher than 7.02% of GPCP-2. ITPCAS still outperformed the other products on a monthly scale. GPCC had a better representation for true rainfall compared to TRMM 3B43 based on r and BIAS values, while TRMM 3B43 had slightly lower RMSE and MAE values. Overall, all evaluation indexes were proximal for GPCC and TRMM 3B43. CMORPH had the poorest estimation of all products on a monthly scale, and GPCP-2 displayed a more accurate capability than CMORPH but a worse performance than ITPCAS, TRMM 3B43, and GPCC.

In terms of temporal scales, all precipitation products were in good agreement on an annual scale, which were superior to that on a monthly scale except for GPCP-2. Meanwhile, there was slightly greater consistency between the products and ground-based measurements in autumn compared to the other seasons.

### The performance differences at individual stations

**Fig 5:**
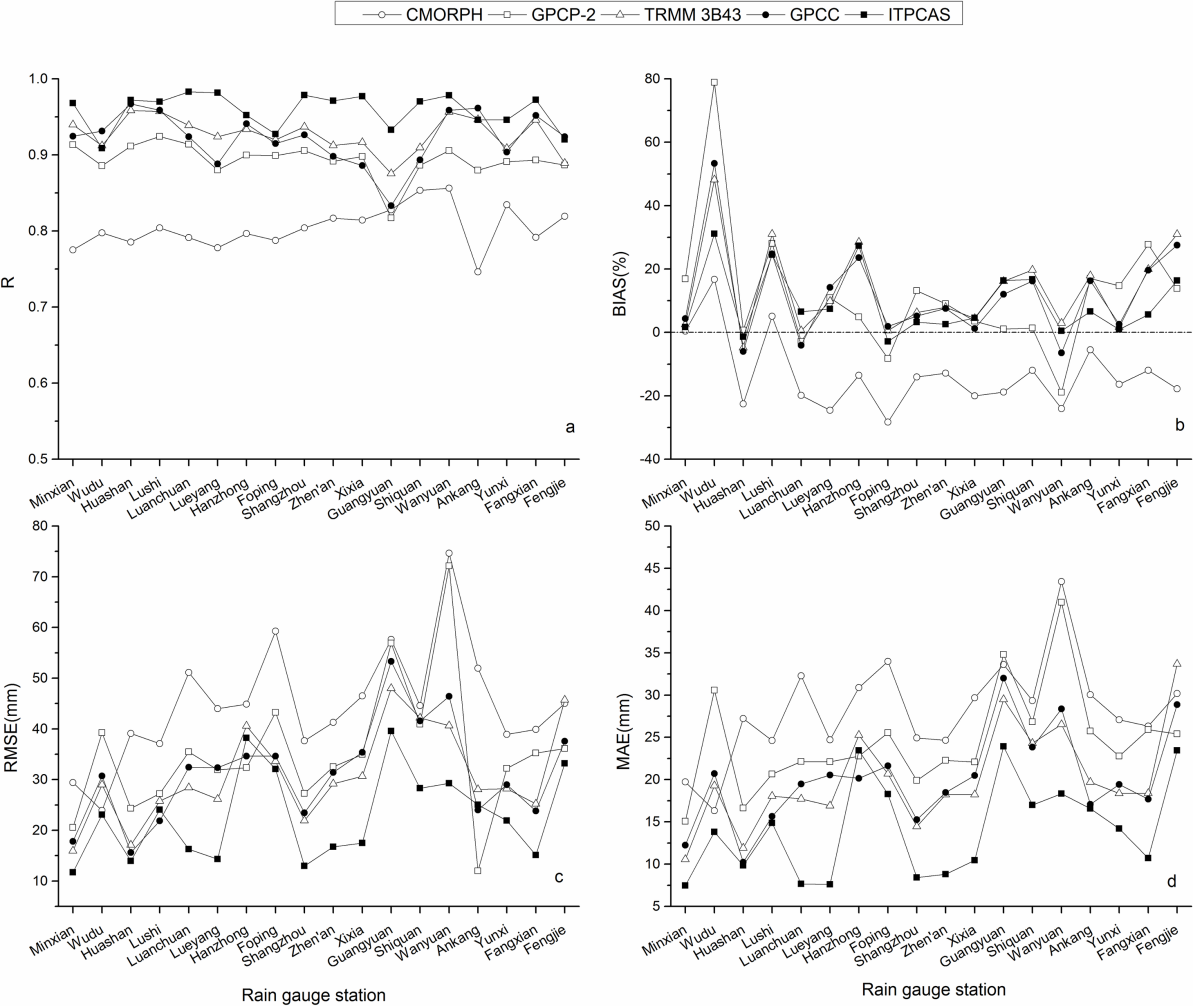
**Evaluation indexes, including (a)R, (b)BIAS, (c)RMSE, (d)MAE, between monthly precipitation from precipitation products and corresponding rain gauge data at individual stations ranged in the order of the station numbers in Table 2** (the station above the dotted line meant overestimation of precipitation, while the station below the dotted line meant underestimation of precipitation).

All rain gauge stations had high r values above 0.7 at the 99% significance level, which are in fairly good agreement with the ground-based measurements (Fig 5A). ITPCAS showed the best performance with a perfect r value of 0.96 followed by TRMM 3B43 and GPCC with average correlation values of 0.93 and 0.92, respectively. GPCP-2 and CMORPH had minor r values of 0.89 and 0.80, which were still fairly consistent with the rain gauge stations.

The BIAS values of 18 rain gauge stations fluctuated in the range of ±30% except for Wudu station, where GPCP-2, TRMM 3B43, GPCC, and ITPCAS had the greatest BIAS (Fig 5B). BIAS of GPCP-2 peaked at a value of 78.85%, which may be attributable to the undulating terrain and uneven rainfall near Wudu station. In addition, CMORPH had negative BIAS values at most gauge sites, except Minxian, Wudu, and Lushi station, which revealed the underestimation of CMORPH in most circumstances. At the same time, the BIAS of CMORPH was farthest from 0 at most stations, meaning it had the largest deviation from the true precipitation.

RMSE values of ITPCAS ranged from 11.71 to 39.55, which were the lowest among the precipitation products suggesting minimal errors (Fig 5C). Meanwhile, RMSE values ranged from 15.97 to 47.98 for TRMM 3B43, from 15.60 to 53.29 for GPCC, from 12 to 72.13 for GPCP-2, and from 23.91 to 74.64 for CMORPH. The greatest RMSE values of ITPCAS, TRMM 3B43, and GPCC were obtained from Guangyuan station, while the highest values of GPCP-2 and CMORPH were obtained from Wanyuan station. Same tendency for MAE was shown in Fig 5D.

Overall, the five precipitation products performed well in terms of obtained r values, with ITPCAS performing the greatest. TRMM 3B43 and GPCC displayed good performances but were slightly inferior to ITPCAS. GPCP-2 had the largest BIAS values at Wudu station. In contrast, CMORPH displayed the lowest r and highest RMSE, MAE, and BIAS values, meaning it had the worst agreement with the observed rainfall data among the five precipitation products.

The 18 rain gauge stations with continuous time series show relatively good agreements with ground-based measurements as a whole based on all evaluation indexes. Although the indexes vary in different regions, they still simulate precipitation reasonably well at respective stations.

## Discussion

### Performance differences at relatively arid and humid years

The total annual precipitation was calculated by the sum of annual precipitation from the 18 rain gauge stations based on a continuous record from year to year. In 2001, a minimum annual precipitation value of 663.84 mm was obtained, while a maximum value of 999.98 mm occurred in 2011. Thus, 2001 and 2011 are regarded as the relatively arid and humid years, respectively, which were about 19.60% lower and 21.10% greater than the average annual precipitation of 825.70 mm. In the following section, the performance differences of the precipitation products in 2001 and 2011 were discussed to further explore their advantages. PD and PPD, mentioned in the Methodology section, were used as the evaluation indexes.

**Fig 6:**
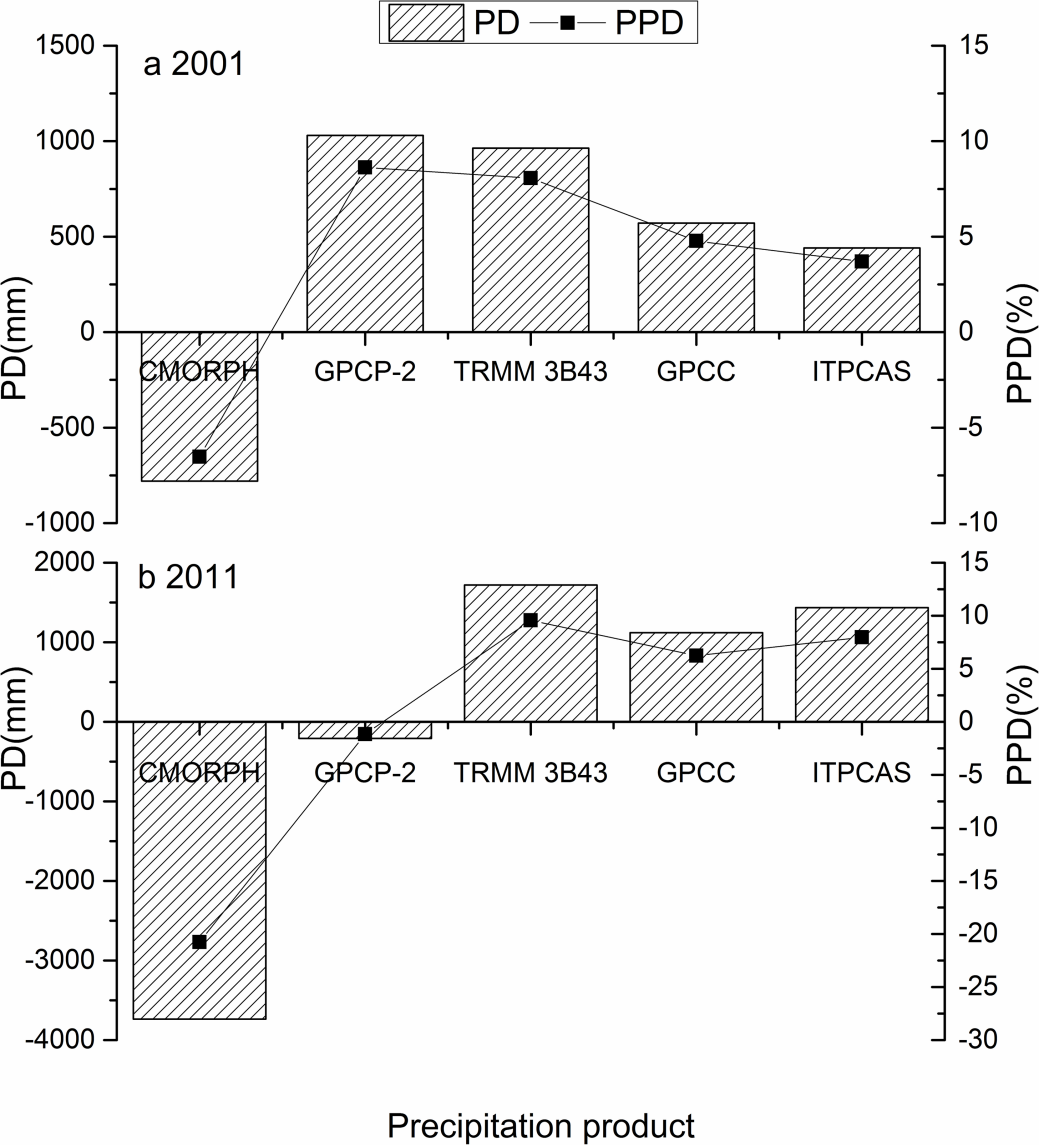
**The PD and PPD for precipitation products in the Qinling-Daba Mountains in (a) 2001, and (b) 2011**.

PPD was used as an example to explain differences in the rain gauge data. In 2001, it appeared that CMORPH had an underestimation of -6.53%, while the other four products showed overestimation in varying degrees for annual precipitation (Fig 6A). Meanwhile, GPCP-2 had the largest PPD with a value of 8.62%. ITPCAS and GPCC performed better than GPCP-2with PPDs of 3.69% and 4.78%, respectively, and TRMM 3B43 and CMORPH were averaged between, which showed that the PPDs of all precipitation products were within 10%. Compared to 2001, the performance of precipitation products in 2011 changed considerably, but PPDs of all products maintained the performance accuracy within 10% except CMORPH with a PPD value of -20.76%, which is a larger negative deviation from the observation data (Fig 6B). Not only did CMORPH continuously underestimate precipitation, but GPCP-2 also showed a minor underestimation with a PPD of -1.16%. In addition, GPCP-2 had the largest PPD value in 2001 and the smallest PPD value in 2011 among all precipitation products. Thus, GPCP-2 was seen as the most unstable product in the relatively arid and humid years. CMORPH proved to be the second most unstable product with PPD values of -6.53% in 2001 and -20.76% in 2011 and also presented the largest fluctuation of -14.23%. GPCC had a PPD of 6.23%, which was slightly better than ITPCAS and TRMM 3B43.

**Fig 7:**
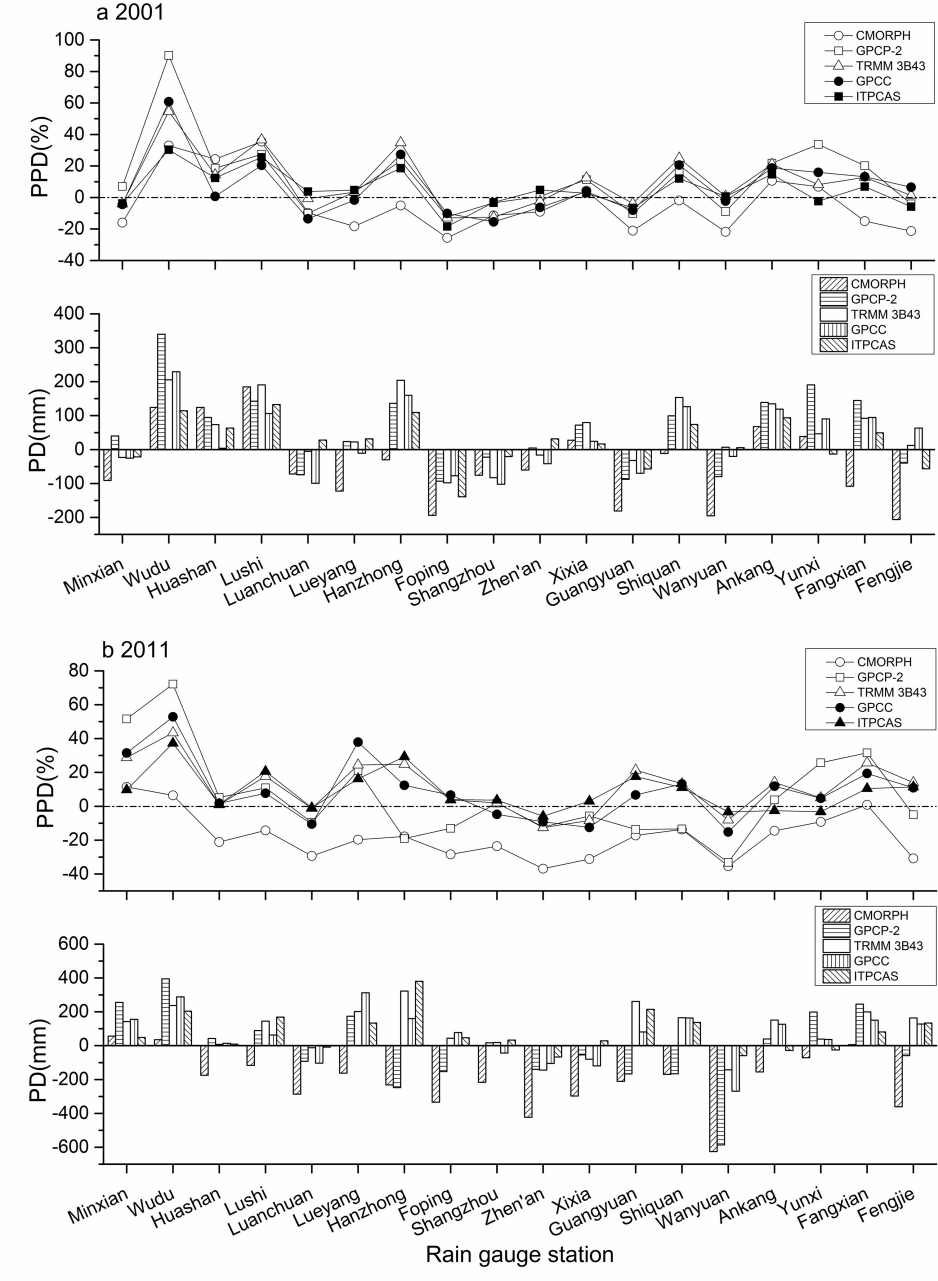
**The PD and PPD at individual rain gauge stations in (a) 2001, and (b) 2011** (the station above the dotted line meant overestimation of precipitation, while the station below the dotted line meant underestimation of precipitation).

After overall evaluation, the performance of each individual station was also assessed in accordance to PD and PPD values (Fig 7).

In the relatively arid and humid years, no obvious trend in PPDs occurred for all rain gauge stations except Wudu, where ITPCAS, TRMM 3B43, GPCC, and GPCP-2 met their maximum PPDs. In 2001, the largest PPDs for ITPCAS, TRMM 3B43, GPCC, and GPCP-2 were 30.33%, 54.55%, 60.78%, and 90.14% and were 37.26%, 43.44%, 52.83%, and 72.19% in 2011, respectively (Fig 7A and 7B). The poor performance at Wudu station is most likely due to the minimal precipitation and undulating topography of this area in the Qinling-Daba Mountains.

### Precipitation gradient

**Fig 8:**
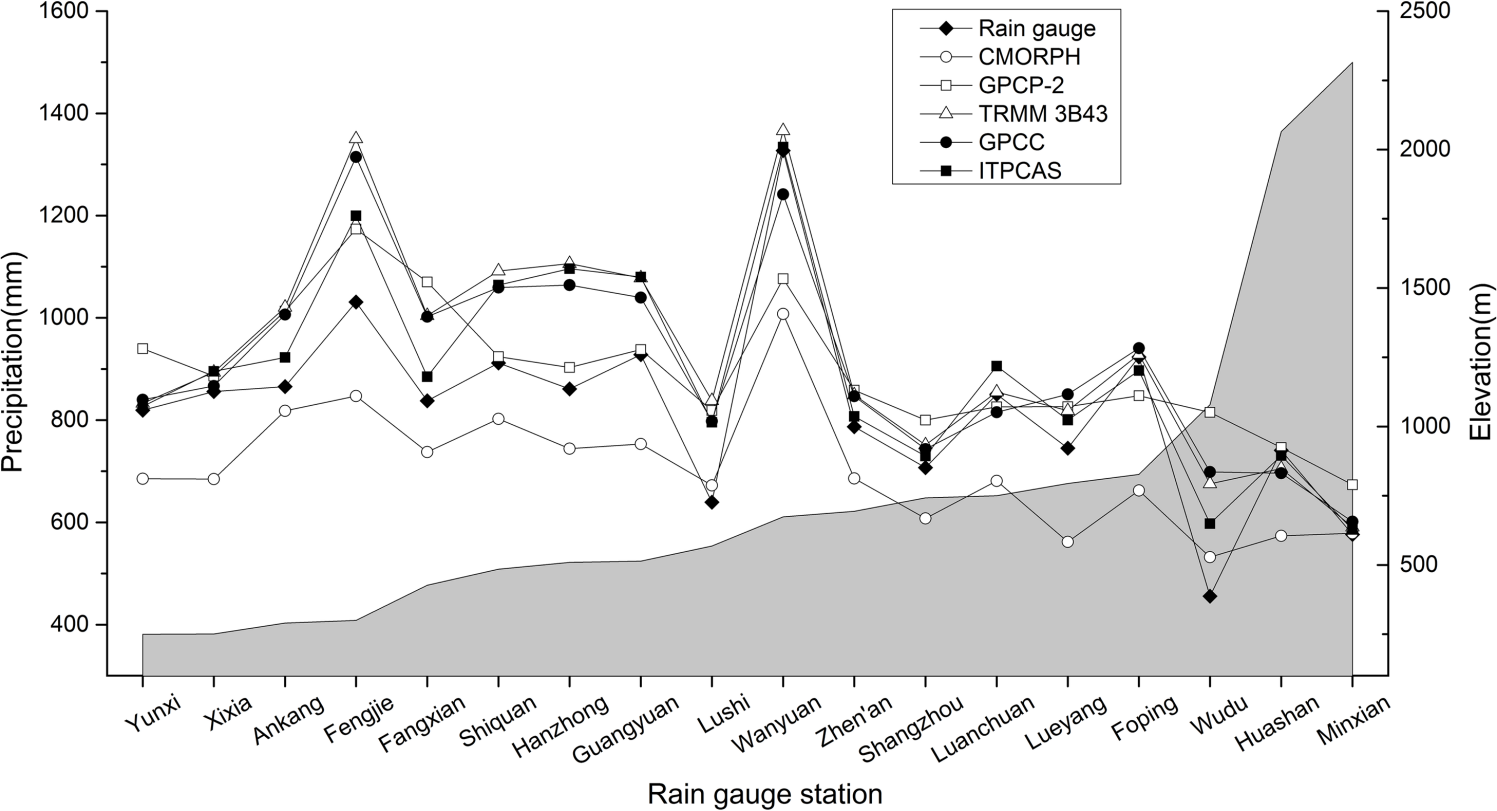
The line chart of annual mean precipitation from observed and precipitation products at increasing altitudes.

The inhomogeneity of precipitation distribution is one factor that caused the diversity in plant population and natural landscape at different elevation zones [47]. Multiple studies on the precipitation gradient in mountainous regions have been conducted [48–51] and suggest that the gradient based on altitude varies with zone, height, aspect, season, and even wet and dry years. To further explore the precipitation variations in the vertical height in the Qinling-Daba Mountains, we analyzed the precipitation gradient of rain gauge precipitation and precipitation products (Fig 8).

**Fig 9:**
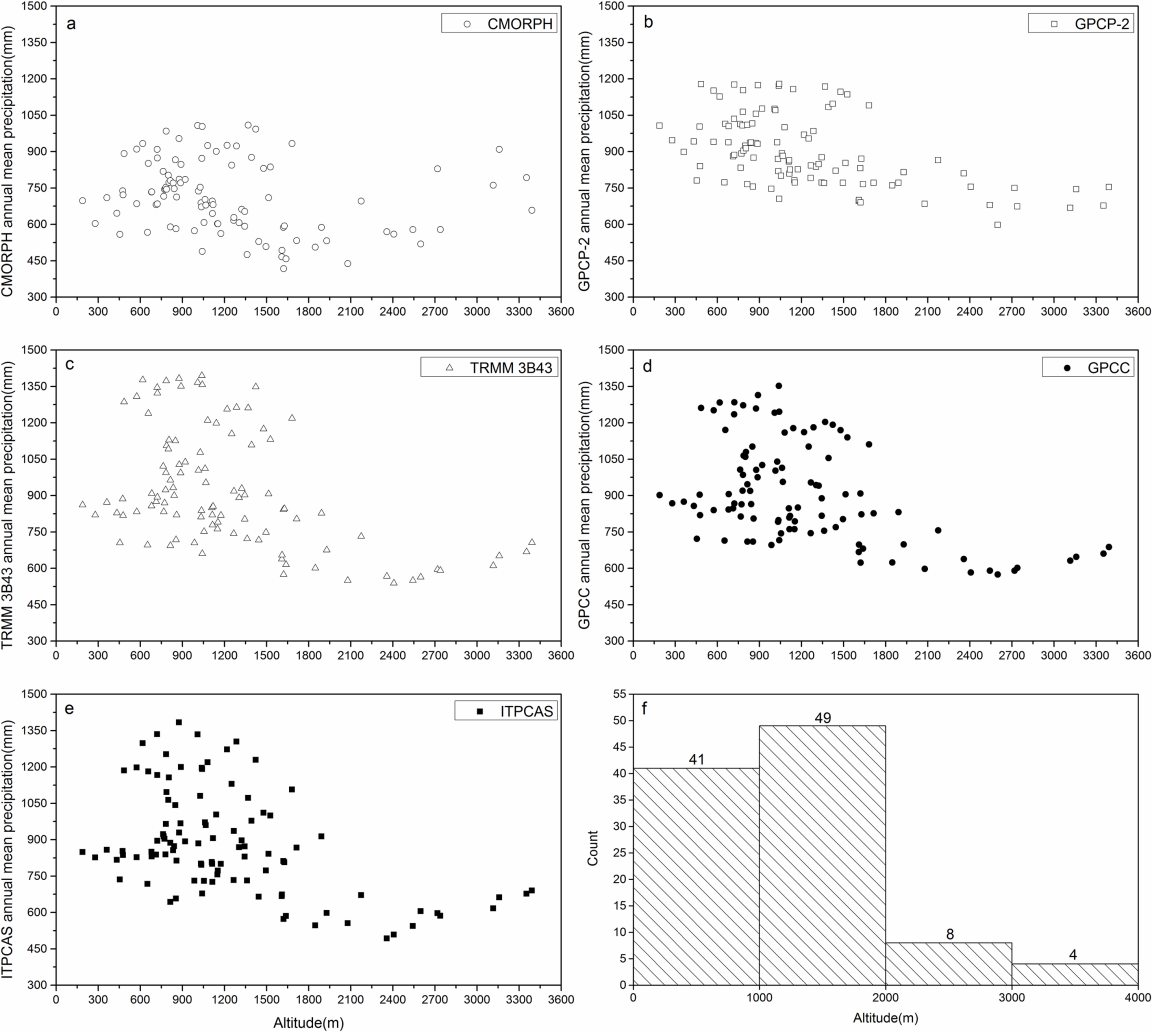
The scatter plots of annual mean precipitation from precipitation products at increasing altitudes. (a) CMORPH, (b) GPCP-2, (c) TRMM 3B43, (d) GPCC, and (e) ITPCAS showed the relationship between the evaluated rainfall from precipitation products and increasing altitudes; (f) showed the grid number at different altitudes.

No clear precipitation gradient can be seen in Fig 8 for both the observed rainfall and the estimated rainfall from the grids where observation site locates in each product, which is different from other existing studies, such as Chen’s research [52] showing a maximum precipitation height of 1400 m and 1900 m for the northern and southern slopes of the Qinling-Daba Mountains, respectively. Considering that only two rain gauge stations are located above 2000 m, one is above 1000 m, and the remaining 15 rain stations are all below 1000 m, it is hard to say that the limited number of sites in higher altitude probably affect the estimated precipitation gradient. To further examine whether the precipitation gradient exists, the relationship between annual rainfall obtained from all grids of each precipitation products and corresponding elevation in the Qinling-Daba Mountains were delineated in Fig 9. There is also no clear precipitation gradient exist in Fig 9. Both Fig 8 and Fig 9 suggest that both existing observation data and precipitation products have no clear precipitation gradient in the Qinling-Daba Mountains.

The precipitation gradient is related to multiple complex factors. Numerous high peaks and fault basins are scattered along the Qinling-Daba Mountains, and the Hanjiang Valley is located between Qinling Mountain and Daba Mountain, which together constitutes the complex terrain and causes uneven distribution of precipitation in the Qinling-Daba Mountains. An obvious precipitation gradient does not exist in this study perhaps due to the diverse spatial distribution of different observation sites and the limited observation data in alpine mountainous areas. There is a possibility that horizontal (latitude/longitude) precipitation gradients dominate in the rain gauge data and precipitation products with relatively low resolution.

On the other hand, the rainfall measured by rain gauge is affected by multiple factors, especially in mountainous areas, where the actual precipitation is often less than the precipitation observed due to undercatch, such as wind effect, wetting and evaporation losses. Yang et al. [53–55] found that the correction factor varied with meteorological conditions and site positions. Thus, the precipitation gradient cannot be examined in the Qinling-Daba Mountains probably affected by ignoring the undercatch without correction. Whether there is precipitation gradient in the Qinling-Daba Mountains needs to further observe in the future.

### Comparison with previous studies

Based on the evaluation of the five precipitation products with ground-based measurements on annual, seasonal, and monthly scales, ITPCAS showed the best performance, TRMM 3B43 and GPCC displayed moderate and similar estimation, GPCP-2 was slightly worse than the first three products, and CMORPH had the worst performance. In this study, the TRMM post real-time research product of 3B43 showed better agreement with observation data than did CMORPH with a higher spatial resolution, which is consistent with previous researches. For example, Hu et al. [56] ranked TRMM 3B43 as the highest among six precipitation products, including TRMM 3B42 RT and 3B43, CMORPH, MWR+ (Global Satellite Mapping of Precipitation Microwave Radiometer plus AMSRU-B), MVK+ (GSMap Moving Vector plus AMSRU-B with Kalman Filter), and PERSIANN over the Ganjiang River Basin, in the Southeast China during 2003–2009. Yang and Luo [57] found that TRMM 3B42 and 3B43 performed better than CMORPH and PERSIANN in the arid region of northwest China. An examination of precipitation products in Thailand by Janjai et al. [58] also suggested that TRMM 3B43 gave the best performance, followed by the GMS (Geostationary Meteorological Satellite) regression model, while CMORPH performed the worst. Pombo et al. [59] pointed out that the rainfall estimates from TRMM 3B43 are more accurate than CMORPH, GPCP 2.2, and PERSIANN in Angola.

In addition, Feidas [60] revealed that TRMM 3B43 is superior to multisource products GPCP and CMAP in a 2.5° × 2.5° grid scale. After comparing CMAP, GPCP, and TRMM products in south China, Huang et al. [61] reported that these three precipitation datasets were able to capture the main features of rainfall amount, while TRMM products showed better capability of simulating the precipitation in the areas with complex terrain. These results are consistent with those we obtained in this study.

As the best product for simulating precipitation on different time scales, ITPCAS was produced by merging TRMM 3B42, GLDAS (Global Land Data Assimilation System), and rainfall observations from meteorological stations in China [33, 62]. It is plausible that the merging rain gauge data is the key factor behind the precipitation dataset’s good performance for accurate rainfall. CMORPH, which is based only on microwave data without fusion with ground-based measurements, showed the worst performance among the five datasets, while ITPCAS most likely merged more gauged stations and gauged precipitation data in China. However, it is difficult to quantitatively define the influence of the number of rain gauge stations and gauged data because we cannot obtain or determine the exact number of gauged stations and gauged precipitation data involved in the five precipitation products in the study area.

These results indicate that various studies and operational practices require diverse spatial and temporal resolutions and that higher and finer resolutions may not always lead to more accurate and expected results for multiple reasons [2]. Sun’s research also showed that GPCP with a 2.5 × 2.5° resolution was superior to CRU (Climatic Research Unit) in a 0.5° × 0.5° grid [63]. No obvious relation has been revealed for spatial resolution with the accuracy of precipitation products in this study, just like CMORPH with the finest spatial resolution.

## Conclusions

Precipitation products from CMORPH, GPCP-2, TRMM 3B43, GPCC, and ITPCAS were evaluated with ground-based measurements on a 0.5° × 0.5° grid and annual, seasonal, and monthly time scales in the Qinling-Daba Mountains during 2000–2014. Performances of the precipitation products in relatively humid and arid years and rainfall variation with altitude were also discussed. Based on the obtained results, the following conclusions were made:

1. ITPCAS has better correspondence with rain gauge data than other rainfall data on annual, seasonal, and monthly scales, which is followed by TRMM 3B43 and GPCC. CMORPH shows the worst agreement with observed data. The precipitation products perform better on an annual scale than on monthly scales. Meanwhile, there is slightly greater consistency between the products and ground-based measurements in autumn compared to the other seasons. All precipitation products suggest good capability at individual stations, but larger errors resulted at Guangyuan, Wanyuan, and Wudu station.
2. ITPCAS, TRMM 3B43, and GPCC presented fairly steady capacity during the relative arid and humid years. Although GPCP-2 and CMORPH show large fluctuations, the percentage precipitation differences (PPDs) fluctuate in the range of 30% in total.
3. All precipitation data, including ground-based measurements do not exhibit significant or obvious precipitation gradients in the Qinling-Daba Mountains, which may be due to limited and scarce rain gauge stations or undercatch in high altitude regions with complex terrain in the studied areas.

According to our study, the accuracy differences between ITPCAS and the other four precipitation products are remarkable. In general, we conclude that out of all examined products, ITPCAS can stimulate precipitation fairly well with the best consistency on annual, seasonal, and monthly scales in the Qinling-Daba Mountains. In addition, further investigations should be conducted on the performance of precipitation products in the future, including the new GPM (Global Precipitation Measurement).
